# Growth Dynamics Explain the Development of Spatiotemporal Burst Activity of Young Cultured Neuronal Networks in Detail

**DOI:** 10.1371/journal.pone.0043352

**Published:** 2012-09-19

**Authors:** Taras A. Gritsun, Joost le Feber, Wim L. C. Rutten

**Affiliations:** Neural Engineering Department, Institute for Biomedical Engineering MIRA, University of Twente, Enschede, The Netherlands; The University of Plymouth, United Kingdom

## Abstract

A typical property of isolated cultured neuronal networks of dissociated rat cortical cells is synchronized spiking, called bursting, starting about one week after plating, when the dissociated cells have sufficiently sent out their neurites and formed enough synaptic connections. This paper is the third in a series of three on simulation models of cultured networks. Our two previous studies [26], [27] have shown that random recurrent network activity models generate intra- and inter-bursting patterns similar to experimental data. The networks were noise or pacemaker-driven and had Izhikevich-neuronal elements with only short-term plastic (STP) synapses (so, no long-term potentiation, LTP, or depression, LTD, was included). However, elevated pre-phases (burst leaders) and after-phases of burst main shapes, that usually arise during the development of the network, were not yet simulated in sufficient detail. This lack of detail may be due to the fact that the random models completely missed network topology .and a growth model. Therefore, the present paper adds, for the first time, a growth model to the activity model, to give the network a time dependent topology and to explain burst shapes in more detail. Again, without LTP or LTD mechanisms. The integrated growth-activity model yielded realistic bursting patterns. The automatic adjustment of various mutually interdependent network parameters is one of the major advantages of our current approach. Spatio-temporal bursting activity was validated against experiment. Depending on network size, wave reverberation mechanisms were seen along the network boundaries, which may explain the generation of phases of elevated firing before and after the main phase of the burst shape.In summary, the results show that adding topology and growth explain burst shapes in great detail and suggest that young networks still lack/do not need LTP or LTD mechanisms.

## Introduction

Studies on developmental changes in biological neuronal networks may advance our understanding of brain development, activity patterns associated to different stages, or the relationship between connectivity and activity. Long-term cultured networks of dissociated rat cortical neurons provide a useful experimental platform for this study [1], [2], [3], [4], [5], [6], [7]. On a relatively long time scale, from several weeks to months in vitro, neuronal cultures undergo major morphological changes in neurite outgrowth and connectivity/topology, with a corresponding impact on activity. Particularly in the first 3 weeks growth is probably a major factor to determine connectivity, whereas at later stages long term plasticity mechanisms may become dominant. A profound feature of activity in cultured cortical networks is bursting; i.e. synchronized firing at an elevated frequency in a large part of the network.

This paper is the third in a series of three on simulation models of cultured networks. Our two previous studies [26], [27] have shown that random recurrent network activity models generate intra- and inter- bursting patterns similar to experimental data. The networks were noise or pacemaker-driven and had Izikevitch-neuronal elements with only short-term plastic (STP) synapses (so, no long-term plasticity, LTP or LTD, included). However, elevated pre-phases (burst leaders) and after-phases of burst main shapes were not yet explained in sufficient detail. This lack of detail may be due to the fact that the random models completely missed network topology and also lacked a growth model, which is essential to study how activity develops with time. The present study adds topology and growth and thereby combines neurite growth models and electrical activity models to predict synchronous bursting behavior, their spatial spread (burst waves) as well as their longitudinal development.

Of course, LTP and LTD mechanisms are widely accepted as essential mechanisms in developing and learning neuronal systems and have been studied experimentally also in cultured networks, as pioneered by Jimbo and co-workers (see [7] for an review], although with limited success. Long term plasticity mechanisms may also largely determine the development of activity patterns, in particular at later developmental stages. However, it remains unclear to what extend the factor growth contributes to the development of network connectivity, in young cultures.

Experimental studies give us a variety of observations ranging from the basic morphological structures of typical cortical neurons like interneurons and pyramidal cells [6], [8], [9] to more specific structures like those of large GABA-ergic interneurons [10]. Analysis of basic cell structures and their growth led to the design of models of neuronal morphogenesis [11], [12] which can be used to build a simulation framework for generating realistic wiring topologies [13], [14]; cell numbers are usually rather limited (for example, N = 62 in [15]). Meanwhile, investigation of more specific neurons underlined their key role in network electrophysiological activity. For example, Voigt et al. [10] showed that large GABA-ergic cells with characteristic extensive neurite arborization and high synaptic density around their somas can drive synchronous oscillatory activity in immature cultured networks (it is to be noted that young GABA-ergic cells start their life as excitatory cells). On the other hand, network topology directly influences network firing. For example, Kitano et al. [16] showed that a network's small-world topology can generate clustered spiking (bursting), where random networks cannot, when using the same electrophysiological parameters. These results suggest that realistic topologies should be implemented into simulations of spiking activity.

A variety of models exist to generate neurite structures (for review see [17]). The focus can be on neurite guidance [18], [19] or on structure formation of the neuritic trees [11], [12]. So far, these aspects have not yet been combined. For example, the generic model of chemotactic based network self-wiring in [19] did not include neurite branching, whereas the model of dendritic outgrowth by Van Pelt and Uylings [12] did not take into account neurite bundling and neurite steering by guidance cues. All these features are critical for the proper formation of synaptic connections in the network, thereby affecting network electrical activity.

In this report we will build a wiring topology for networks of up to 50,000 neurons, using the model of neuronal morphogenesis by Van Pelt and Uylings [12]. Subsequently, this wiring layout is used as the connectivity matrix in a network model to generate spiking activity. As an extension, we will add the neurite guidance model by Segev and Ben-Jacob [19] to mimic axono-somatic targeting. Finally, to both models we will add a set of fast growing interneurons to mimic large GABA-ergic neurons. Together, this leads to four model variants. Simulation results will be characterized as much as possible by the same intra- & inter- burst parameters as used before in our experimental studies, for comparison and evaluation of models with experimental data. Networks will be followed until 22 days in vitro (DIV). The automatic adjustment of various mutually interdependent network parameters is one of the major advantages of our current approach.

The purpose of the model is to show how axono-somatic targeted-growth networks develop into consistently repetitive burst producing systems, as observed in experiment. With burst characterization both in time (burst shapes; burst rhythms) and in space (burst propagation, burst “waves”), with variable network size. The model also will enable to investigate propagation of bursts along the network boundaries (reverberation effects), in the spatial domain as well in its temporal utterance in the pre- and after-phases of burst shapes. As such, it will also contribute to answer the important general question to what extend growth may explain recorded activity patterns during the first 3 weeks in sufficient detail.

## Methods

### Simulation of network morphology

In the present study we proceeded on the research by Van Pelt and Uylings [12] who developed a stochastic approach for neurite outgrowth models. In further research Koene et al. [13] used their ideas to simulate network topologies and observe network development. Their modeled structures for cell types containing both cortical pyramidal neurons and interneurons as well as their development in time, were successfully compared with experimental data, e.g. [12], [20]. We used their validated functions of neurite elongation and branching rates as well as their model parameter values, so that all simulated neurites grew at the same rates as in cultured cortical neurons. For the time development of the network topology we used a time step of 1 day, as from experiment it is known that neurites do branch not more than once a day [12].

**Figure 1:**
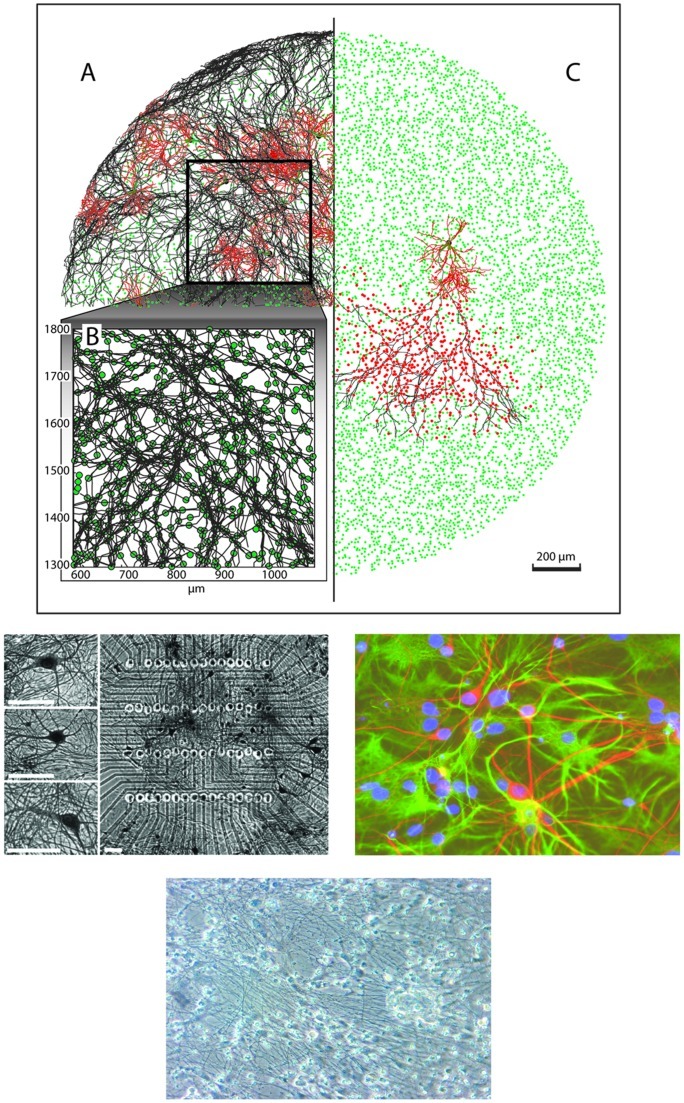
Simulation of neurite morphology in the field-guided network of 10,000 neurons. A: The neuronal somas are indicated in green. For 0.5% of these neurons, marked with a large black dot, the neurite structures are shown: axons (black) and dendrites (red). B: Close-up of A, showing bundles of axons that occur in field-guided growth models. C: Axon (black line) and dendritic tree (red lines) of a pyramidal neuron (large black dot). Neurons that receive input from this pyramidal neuron are indicated by blue dots. D: Illustration of parallelism of neurites, observed in G.Gross'lab. A neuronal culture _right_ over a microelectrode array. The uniform grid of points shows electrodes where neuronal activity is measured. Left panels show individual neurons. White bars are 50 _µm _center-to-center distances between electrodes is 40 _µm_. Taken from [46]. E: Some parallel tracks (e.g. the red fibers running under 45 degrees at the right half of the photo) can be observed in this graph. Immunostaining micrograph of neuronal network cultured at 12 DIV on MEAs. Neurons were stained with antibodies to MAP2 (green) and Neurofilament 200 (NFH,red), respectively. Cell nuclei were stained with Hoechst 33342 (blue). Bar = 40 um. Courtesy T. Ugniwenko, University of Kaiserslautern. F: Massive parallelism in a culture at 14 DIV, University of Twente, prepared as described in methods.

We designed circular networks, incorporating 10,000–50,000 neurons with homogeneous (uniform) density of about 2500 neurons per mm^2^. The average radius of these virtual cultures varied from about 1.1 to 2.5 mm. We assumed that somas adhere firmly to the substrate. The neurites grew planarly and were allowed to cross each other. We used a triangular lattice (of 85,000 to 433,000 lattice cells) in a circular area to randomly arrange neurons. Every soma was represented by a stationary (not moving) unit occupying one of the lattice cells. Circular shaped somas, with radius 6.25 µm, were placed in the center of a lattice cells with random shifts of ±5 µm. We took *m* = 20 µm for the edge length of the lattice, yielding only small overlap for excentrically placed cells with a neighbor (if present, see inset of figure 1). Neurite outgrowth always remained within the predefined circular space (unless mentioned otherwise).

We employed two models of growth, random and chemotaxis-guided. The random modele (Koene et al. [13]) identified the direction vector of the neurite outgrowth as the sum of vectors preceding it plus a random perturbation (*r_a_*) which was drawn from a uniform distribution over the interval [a_min_, a_max_]. The guided one applied a chemotactic neurite navigation approach presented by Segev and Ben-Jacob [19] to calculate the final direction of axonal outgrowth. From the first day in vitro (DIV) plated neurons (somas) release chemo-attractive cues guiding axonal outgrowth. Growth cones sense a gradient of guidance cues and alter their direction in the next simulation step. Instead of random perturbation (*r_a_*) we calculated the directional derivative 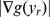 of the soma concentration over all the local neurons *y_r_* within a circular searching area with radius *d_x,y_*, set equal to the axonal elongation *Δl*. Here *g* satisfied the following diffusion equation: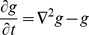. Taking into account the cytoskeleton rigidity and membrane tension structure in a consolidated axon we also defined the same bounds [a_min_, a_max_] for 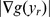. If there were no neurons within the local searching area (*g(y_r_) = 0*), axons did not change direction but continued growing in the same direction.

The two approaches described above will be referred to as *randomly growing* and *field -guided* networks, respectively. On the first virtual day in vitro (vDIV) axons elongated with random perturbations in both approaches. Starting from the second vDIV, axons extended towards higher spatial somatic concentrations in field-guided networks Then from the 3^rd^ vDIV, growing axons coming across dendrites defined the set of ‘candidate synaptic connections’. The successful synapses were randomly chosen from all the temporal candidates at every subsequent vDIV with a probability of 0.05. This probability was chosen to adjust the number of generated synapses per neuron to the experimental range [21].

For transmission delays we adopted the results from a modeling study by Manor et al. [22]. They considered simple cases where the geometrical ratio of neurites (i.e. axons) was always equal (set to 1), meaning that the sum of the diameters of the daughter branches was the same as the diameter of the parent branch. Action potentials traveled with a constant velocity of 540 µm/ms in the root branch. With this propagation speed, the axonal delay for an axonal length of about 3.5 mm on vDIV 21 corresponded to an average propagation delay of 6.5 ms. According to [22], these axonal delays make up about 72% of the total delay. The remaining 28% was added to account for the synaptic and dendritic delays (fixed at 2.5 ms). These values were confirmed in two other experimental studies using cultured cortical networks [23], [24].

To obtain initial values for absolute synaptic weights we followed a study by Williams and Stuart [25] who investigated how post-synaptic potentials depended on the distance from the soma to the location of the synapse on the dendrites. They found that the amplitude of somatic post-synaptic potentials decreased with the distance to the soma. We implemented their finding by defining synaptic strengths with an initial PSP height of 1 mV, that linearly decreased with the distance to the soma by a factor 0.0025/µm. As most network parameters now directly depended on the topology structure, we did not have to set them manually, like we did in previous models [26], [27]. Thus, axons contributed to transmission delays only, whereas dendrites also contributed to somatic post-synaptic potential attenuation.

### Characterization of the network topology

To analyze structural properties of the simulated network topology we calculated the characteristic path length (*L*) and the clustering coefficient (*C*) introduced by Watts and Strogatz [28]. *l_ij_* between two neurons is defined as a minimum number of synapses through which the action potential has to travel to get from one neuron to another. *c_i_* indicates the number of connections between all neurons connected to chosen neuron *i* as a fraction of the maximum possible number of such connections. It is defined as:
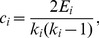
(1)where *k_i_* is the degree of neuron *i* (i.e. number of synaptic connections with different neurons) and *E_i_* is the number of connections of neuron *i*. *L* and *C* are calculated as the averages of *l_ij_* and *c_i_*, respectively. (This was possible only for networks of 10,000 neurons because of computational limitations.). To acquire statistical description of the generated topological structures we employed a quantitative measure of ‘small-world-ness’ (*S*) introduced by Humphries and Gurney [29]: First, using the analytical approximation by Newman et al. [30] we estimated the average *L_rand_* and *C_rand_* for (Poisson) random graphs with the same number of neurons (nodes, *n*) and average number of connections per neuron (average degree, *<k>*) as in simulated networks.
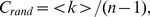
(2)

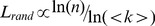
(3)Then *S* was calculated as following:
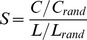
(4)


At the end of each vDIV (corresponding to realistic neurite outgrowth observed on one day in neuronal culture [12], [20]) the connectivity matrix was built out of all successful synapses. This matrix entered the spiking model which then simulated 1 hour of spiking activity.

### Simulation of spiking activity

To simulate the firing activity we employed the (pulse-coupled) spiking neural network model as described in [26], [27]. The system of equations that networks generate can be presented in the following way:
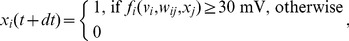
(5)where
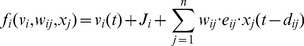
(6)


Here, *x_i_* is the firing pattern of a receiving neuron *i*, *dt* is the simulation step and *x_j_(t -d_ij_)* represents the state of a transmitting neuron *j* (*i,j* = 1, 2,… n, where *n* is the total number of neurons). Synaptic noise, independent for different *i's* is presented by *J_i_*. In our simulations each neuron received a Poissonian spike train (*J*) of rectangular 1 ms pulses, with a mean rate of 80 Hz and with an amplitude normally distributed between 0 and 8 mV, as described in [26], [27]. The variable *v_i_(t)* characterizes the dynamics of the neuronal membrane potential, as described by Izhikevich [31]; *e_ij_* = −1 for inhibitory neurons and +1 for excitatory neurons. *d_ij_* is the total transmission delay that incorporates axonal synaptic and dendritic propagation latencies as described before. *w_ij_* is the momentary synaptic weight obtained from the phenomenological model for short-term plasticity [32]. Parameter values for the Izhikevich neuronal models were set randomly (using a normal distribution, see [26]) in ranges taken from [31], such that our networks contained 3 types of excitatory neurons (80% of total, including regular spiking, intrinsically bursting and chattering neurons) and two inhibitory types (including fast spiking and low-threshold spiking interneurons). We applied parameter values obtained by Gupta, Markram et al [32], [33] to describe the phenomenological synaptic model of short-term plasticity. These were the same as used before (for a summary see Appendix S1).

In this study, we focused on the effect of network topology and its development on the resulting activity patterns. We used a set of neuronal parameters that adequately reproduced the dynamics of cortical neurons [31]. This set contained a mixture of all neuronal cell types that exist in the cortex, which gave our simulations a certain degree of robustness against variations of cell properties. However, it should be noted that our results might be affected by changes in cell properties. Simulations of network spiking activity were implemented in *C*, using Euler's forward method for numerical integration of differential equations with a simulation time step of 1 ms.

### Characterization of bursting activity

To validate the simulated data against experiment, the layout of 60 micro-electrode arrays (MEAs, see under *Cultured networks*) was mapped onto the network topological space to pick up spiking activity around their virtual electrodes. To obtain a robust set of data, we used 100 of such mappings for each simulation. Network bursts were calculated as the total firing rate (firings at all virtual electrodes summed) from the mapped array as is also done for experimental recordings [2]. We also used their burst definition i.e. a burst was detected when two or more spikes were detected for each active virtual electrode (with spike rate >0.1 Hz) within 10 ms bins. Burst profiles were smoothed using a Gaussian filter with 5 ms. Maximal Firing Rate (mFr) is the firing rate at the peak of the burst profile. Inter-burst intervals (IBIs) were calculated as the time intervals between peaks of neighboring bursts. A superburst was defined as a series of bursts with IBIs ≤1 s.

In addition, we will present spike raster plots showing all simulated neurons. We applied a (counter-clockwise) spiral order for neuronal indexing, such that low index neurons were at the center of the network.

### Cultured networks

Experimental techniques such as culturing and recording are explained in detail in [2]. In short, MEAs had 60 Titanium-Nitride electrodes (with 30 µm diameter) in an 8 by 8 square grid with 200 µm electrode spacing. MEAs were coated with poly-ethylene-imine to increase adhesion. Cortical cells (taken from the entire cortex) were obtained from newborn Wistar rats. The dissociated cells were plated at a concentration of 10^6^ cells/ml, and allowed to adhere for 2 hours. Then the non-adhering cells were removed by refreshing the medium which was changed twice a week. The resulting monolayer had an initial density of about 5000 cells/mm^2^, gradually decreasing to ∼2500 cells/mm^2^ after 2 weeks The total plated area is about 100 mm^2^, so with 250000 cells, at last. The MEA's electrode area of approximately 2.5 mm^2^ comprises about 12500 to 6250 cells. The cultures were stored in an incubator at 37°C, 5% CO_2_ and near 100% humidity. Custom made LabView (National Instruments, Austin, Tx) programs were used to control data acquisition.

## Results

### Neuronal morphology and network topology

Both *field-guided growing networks* (Figure 1 A, B, C) and *randomly growing networks* (Figure S1) generated quite complex neurite topologies for our large scale networks of 10,000–50,000 neurons. In the guided models, axons grow towards locations with relatively high somatic density. We observed that axons tended to grow “in parallel”, which became more recognizable in longer simulations. Figure 1A shows the neurite structures of 0.5% (randomly chosen) of the neurons (note some bundles of axonal trunks in close up, fig. 1B). While neurite trees grew with the course of time, axons encountered dendrites of other neurons and generated local connections. Figure 1C shows the neurite structure of a typical pyramidal neuron (black), its axon (black) and dendrites (red) and the somas of its postsynaptic neurons (red) after 21 vDIV. Figures 1D, E, F show parallel growth features in experimental cultures from three different labs, older than the1^st^ week in vitro.

**Figure 2:**
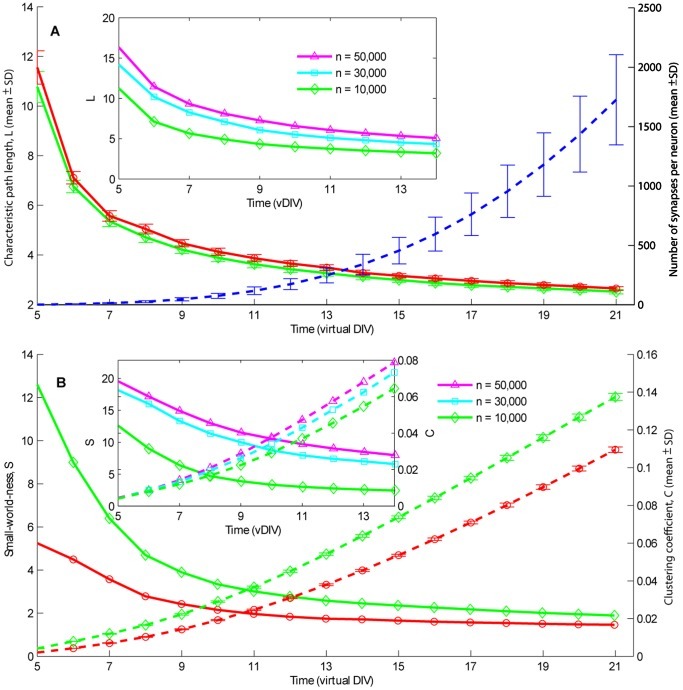
Evaluation of the network topology development during the first 3 virtual weeks. A: The dashed line depicts how the number of synapses per neuron (i.e. the connectivity) increased during aging (mean ±SD; right axis) The number of synapses did not depend on the network size. Solid lines show how the characteristic path length (*L*) (left axis) decreased during aging in field-guided networks with large GABA-ergic neurons (green ◊) and networks without such neurons (red ○). The inset shows *L* versus time for field-guided networks with sizes n = 10,000 (green ◊, as shown in the main panel); 30,000 (cyan □) and 50,000 (magenta Δ) neurons. Error bars represent the standard deviation of 5 network realizations. B: Development of the clustering coefficient (C: dashed lines; right axis) and “Small-world-ness” (S: solid lines; left axis) in field-guided networks with (green ◊) and without (red ○) large GABA-ergic neurons. The inset shows *C* and *S* for field-guided networks with n = 10,000 (green ◊); 30,000 (cyan □) and 50,000 (magenta Δ) neurons. S is a relative measure of the topological similarity between given and random (Poisson-distributed) network graphs. Random network graphs have S∼1, whereas higher S values indicate small-world networks. Both network types gradually developed from small-world to almost random. At early stages of development large GABA-ergic neurons had more influence on “small-world-ness” than at later stages where both network types approached the graph structures of random networks.

While the number of synapses per neuron, referred to as connectivity, increased (Fig. 2A, blue dashed line), the neurons connected to more neurons, at longer metric distances. This resulted in shortening of the characteristic path length (*L*) (Fig. 2A, red and green curves, each averaged over 5 network realizations), and less unreachable neurons.

Voigt et al. [10] showed that cultured cortical networks may contain about 5% fast-growing neurons (large GABA-ergic neurons) which play an excitatory role in early developmental stages. To mimic those neurons, we doubled the neurite elongation rate in 5% of the randomly chosen neurons and inverted the negative weights of their synapses. Their longer neurites created connection shortcuts between neurons at relatively large metric distance, which further increased the percentage of reachable neurons and decreased the characteristic path length *L* (see eq. 3; Fig. 2A, green curve), compared to networks without such neurons at corresponding age (Fig. 2A, red curve). Larger networks had higher values of *L*, as shown in the inset of Figure 2 A

Figure 2B shows developmental curves (each averaged over 5 network realizations) of the clustering coefficient (*C*) and “small-world-ness” (*S*), see equations (2) and (4). In all models *C* slowly increased with time. Figure 2B shows 2 typical examples of the development of *C* (dashed lines) and *S* (solid lines) in field-guided networks with (red) and without large GABA-ergic neurons (green). Randomly growing networks showed very similar values of *L*, *C* and *S* (not shown here). Compared with other neurons, large GABA-ergic neurons had more extensive neurite arborizations, so they were superior in interconnecting nearby neurons. A relatively higher *C* as the result of more interconnected neighborhoods also suggests that large GABA-ergic neurons may facilitate effective generation of a small-world topology.

When *<k>* ∼1, the estimated average path length *L_rand_* converges to ∞. Therefore, *S* shows relatively high values shortly after the onset of network formation. When first connections were formed, *S* had high values (peak around 170 at vDIV 4). At this early stage of topology development networks usually had small sets of chain-linked neurons with *L* = 10 (without large GABA-ergic neurons) or *L* = 30 (with large GABA-ergic neurons). After its peak, *S* decreased exponentially with time as shown in Figure 2 B. As *S* was always higher than 1, all generated topologies were classified as small-world according to definition in [29]. As expected, *S* was always higher in networks with large GABA-ergic neurons than without. Larger networks had higher values of *C* and *S* as shown in inset of Figure 2B.

### Development of bursting activity

**Figure 3:**
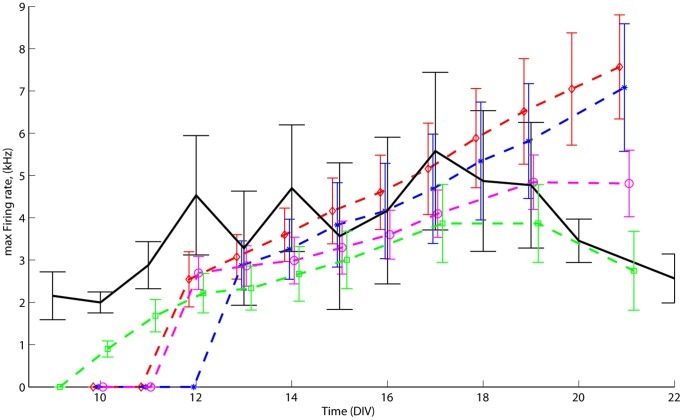
Development of the maximal firing rate (mFr) during bursts. mFr's (means ± SD) are displayed for the 4 model types (dashed lines), and for experimental data, averaged over 5 cortical cultures (black solid line). Randomly growing networks with (magenta ○) and without large GABA-ergic neurons (red ◊), as well as field-guided networks with (green □) and without large GABA-ergic neurons (blue •) are presented. In the presence of large GABA-ergic neurons field-guided networks started bursting 3 vDIVs earlier. For clarity, mean values and error bars representing standard deviations at a certain day were slightly shifted to avoid overlap. Typical examples of simulated and experimental burst profiles are shown in Figure 6.

We examined four different models: *randomly growing* and *field-guided* network models, both *with* and *without* large GABA-ergic neurons. For each type we generated up to 3 different realizations of growing networks. In this paragraph we show results from one of the network realizations for each model type. Other realizations showed similar development of intra- and inter- burst parameters. Different realizations of the networks had slightly different numbers of the various neuronal types, but that did not influence the final outcome. Spiking activity was simulated for 1 hour per virtual day for 3 virtual weeks. At every vDIV we applied a burst detection algorithm on spike sequences, acquired at 100 differently mapped virtual micro-electrode array (MEA) layouts (each with 60 electrodes, see methods). The starting point of bursting activity was identified whenever the algorithm detected the first burst. As soon as bursting started, of the maximum firing rate mFr could be calculated. In field-guided networks, the presence of large GABA-ergic neurons resulted in a 3 vDIVs earlier onset of bursting (green: vDIV 10 vs. blue: vDIV 13), whereas it did not affect the first day of bursting in randomly growing networks (magenta and red: vDIV 12). Figure 3 shows the average development of mFr during bursts in the 4 model types (dashed lines), as well as an experimentally determined average (solid line) of 5 cortical networks presented in [18]. Figure 3 shows a common trend in experimental data of increasing mFr during the 2^nd^ and at the beginning of the 3^rd^ week and decreasing or stabilizing mFr thereafter. A similar trend was only observed in networks with large GABA-ergic neurons (green and magenta).

At later stages of development (2nd half of 3rd virtual week) in the models with large GABA-ergic neurons, bursting activity was dominated by superbursts giving the network relatively shorter time (less than 100 ms) to recover (i.e. elevation of the neuronal membrane potential from reset to resting membrane potential). Therefore during superbursts networks could recruit fewer neurons which resulted in stabilizing mFr during the subsequent bursts.

**Figure 4:**
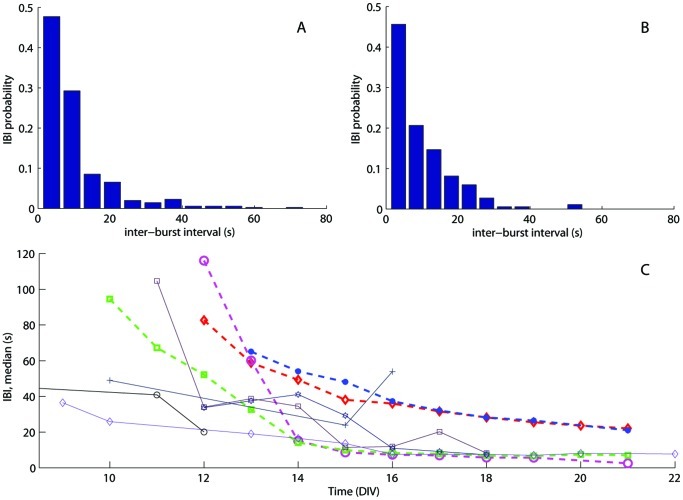
Comparison of inter-burst intervals in simulations and experiments. Typical examples of experimental (A) and simulated (B) IBI histograms constructed from 1 h samples with 355 and 373 bursts respectively, both acquired at 18 DIV. The simulated IBI histogram was generated with a field-guided network of 10,000 neurons with large GABA-ergic neurons. The three other model types had very similar IBI distributions. Bin size was calculated according to [43]. (C): Development of IBI median for experiments, acquired from 5 cortical cultures (thin solid lines) and simulated data (thick dashed lines). Randomly growing networks with (magenta ○) and without large GABA-ergic neurons (red ◊) as well as field-guided networks with (green □) and without large GABA-ergic neurons (blue •) are shown.

For every vDIV we calculated inter burst intervals (IBIs). Both in experimental and simulated data IBIs had characteristic skewed distributions. Figures 4A and B show typical examples based on 1 h samples at 18 DIV from recorded data (4A) and simulated data with a field-guided network with large GABA-ergic neurons (4B). Figure 4C shows IBI median curves acquired from 5 cultures (solid lines) and 4 simulations of different network types (dashed lines). The majority of experiments and simulations share a common tendency of shortening IBIs over time.

### Spatial activity propagation (burst waves) and boundary reverberation

**Figure 5:**
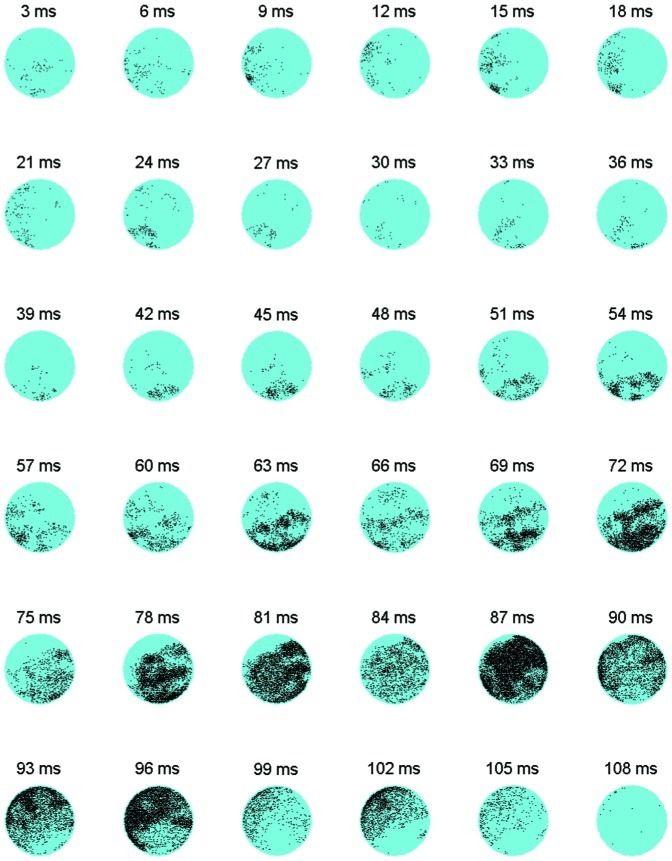
Spatio-temporal propagation of a network burst. Example of a propagating burst in a field-guided model with large GABA-ergic neurons, presented as a snapshot sequence. The time difference between two consecutive snapshots is 3 ms. Each snapshot shows the spatial locations that produced at least one action potential in the last 3 ms (yellow dots). Particularly “reverberating” activity against the network borders was observed in the time window from 63 to 102 ms. N = 10,000 neurons.

In this section we describe the spatiotemporal spiking patterns generated by the network models. In all simulations we observed a wave-like propagation with “reverberating” activity (along the circular network borders) as shown in Figure 5.

**Figure 6:**
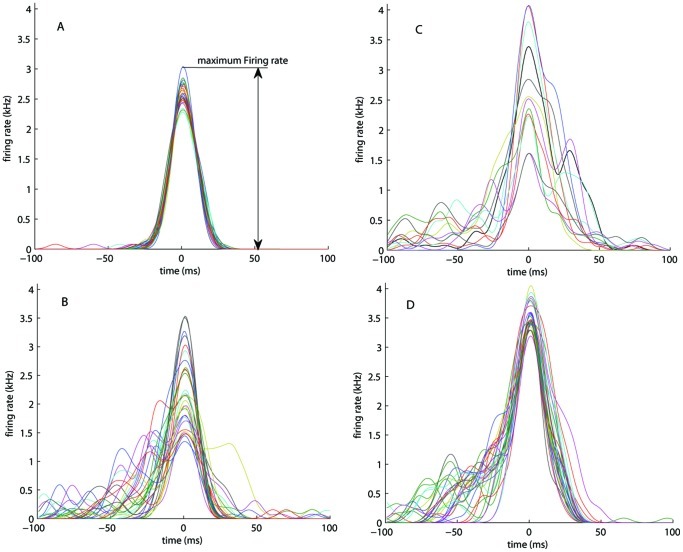
Typical network burst profiles. Networks consisted of 10,000 (A and B) or 50,000 neurons (C) and were wired using the field-guided approach with large GABA-ergic neurons. D: example of experimental bursts taken from previous study (culture # 4) [14].Bursts were detected in the recorded or simulated activity acquired from the networks of the same (virtual) age (12^th^ vDIV for A and C, and 19^th^ (v)DIV for B and D), and aligned by their peaks.

We noticed that bursts started spontaneously at random places or local areas and then slowly recruited neighboring regions. This relatively slow recruitment appeared as pre-burst phases in network burst shapes (spike rate versus time). Upon sufficient recruitment the bursts quickly propagated as waves through the whole network leaving behind a pool of “depressed neurons” which were characterized by a membrane potential value below the resting potential. During bursts, spiking activity was usually higher at the network borders, creating a notable reverberating wave along the network boundary. With given speed of the propagating wave and neuronal recovery, in larger networks, where the traveling wave has to cross a longer distance, the pool of depressed neurons should have more time to recover before the wave fades out. We tested this assumption in one realization of a network model with 50,000 neurons. Simulations of larger networks indeed showed numerous local after-waves raising and fading for brief periods (of 10 ms). The pool of recovered neurons in larger networks frequently showed activity after the main wave. Local after-waves left a print on burst profiles as low-firing post-burst phases. Pre- and post-burst phases were highly variable, as also observed in experimental data [16]. In contrast, smaller networks (of 10,000 neurons) very rarely generated post-burst phases, or highly variable pre-burst phases. Only 2 out of 6 smaller networks showed post-burst phases at the end of the 3^rd^ virtual week (around 19 vDIV) whereas larger networks showed similar results 7 vDIVs earlier. Figure 6 shows typical examples of burst profiles from network models with 10,000 neurons at 12 and 19 vDIVs (A and B respectively), and 50,000 neurons at 12 vDIV (C), as well as an example of experimental bursts at 19 DIV (D) taken from a previous study [16].

**Figure 7:**
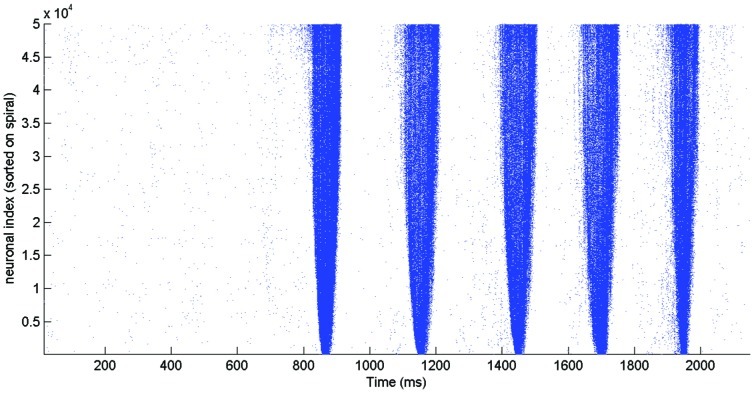
Spiking activity of all the neurons. Spike raster generated by a field-guided network of 50,000 neurons with large GABA-ergic neurons. Note that a spiral order for neuronal indexing has been used, such that low index neurons are at the center of the network. This figure shows that bursts start in neurons with high indices, i.e. neurons located on the edge of the network.

We also found that networks with large GABA-ergic neurons could produce series of bursts with IBIs shorter than 1 s, referred to as superbursts. These networks showed firing between the bursts in chain-like series of events, providing more time (and additional synaptic input) for neuronal recovery. When feedbacks along the network border were activated, these networks produced the next burst shortly after the previous one (1 sec or less). Figure 7 shows a raster plot of a super burst generated by a field-guided network of 50,000 cells with large GABA-ergic neurons. To verify that feedback along the border around the network facilitates super-bursts, we ran several simulations without an outgrowth restricting circular border, i.e. nothing confined neurite outgrowth beyond the network area. Such networks did not generate IBIs ≤1 s, while the intra- and inter- burst parameters were similar to those in networks with border restricted outgrowth as described above. However, the shortest IBIs were about 2 sec.

## Discussion

We employed large scale networks of 10,000–50,000 neurons to analyze bursting activity generated by two sequentially combined models. In the first model growing neurons formed a self-wired topology which yielded network parameters that were entered into the second model to generate spiking activity. This approach can be used for studying network behavior at different levels ranging from spiking activity patterns of individual neurons or local circuits to more global assemblies showing synchronous bursting. In the present study we focused on network bursting as perceived experimentally by mapping the 60-electrodes MEA layout onto the network space. To our knowledge, such a combination of models has never been reported before. In a recent study by Tetzlaff et al. neuronal avalanche development was simulated with an alternative network model where neurites are represented as probability zones and connectivity is derived from the overlap of these zones [34]. Their model demonstrates the interplay between activity and connectivity which our model is not capable of. However, the topology of the network is not produced by Tetzlaff's model; for that, a more detailed description of the neurite outgrowth would be necessary.

The automatic adjustment of various mutually interdependent network parameters is one of the major advantages of our current approach. In fact, connectivity, axonal delays and synaptic strengths are linked together by the neurite maps of individual neurons. The connectivity parameter (number of synapses per neuron) depends on the spatial distance between axons and dendrites of pre- and post-synaptic neurons, respectively. Delays and synaptic strengths depend on axonal length and the distance from the synapse to the soma, respectively. These relationships exist only in networks where connectivity is an emergent property of neuronal morphology. Therefore, given realistic parameters for generating neurite maps, a network presumably acquires a more realistic connective topology. This differs completely from commonly employed methods such as random wiring of synaptic connections [16], [26], [27]. Moreover, the growth model introduces the “dynamic” developmental time dimension into network modeling, allowing also comparison to development of activity during long-term culturing. As stated above (see Methods, development of network morphology) we used a 1–day time step in the growth development. As an extra check, this 1-day step was compared against smaller steps of 6 and 12 hours; these steps gave the same results as the 1-day step.

### Morphology and activity development

During axonal outgrowth neurons connected to an exponentially increasing number of other neurons. This led to an increased connection probability between two random neurons, and resulted on average in a lower number of intermediate neurons between two not directly connected neurons, thus decreasing the average graph distance. Furthermore, at each subsequent vDIV network graphs acquired more interconnected neighborhoods as indicated by an increased *C*. All created topological structures were characterized as small-world graphs, and *S* showed values comparable to other network examples (e.g. in neural network of C.elegans *S* = 4.51) [29]. The small-world-ness (*S*) scales up with increasing network size as indicated by Humphries and Gurney [29] and confirmed by our simulations (Fig. 2B inset). Following the definition of Humphries and Gurney, the topology of networks with connectivity equivalent to mature cultures (3 weeks and older) is close to random as *S* approaches 1. In the first virtual week, groups of nearby neurons formed local circuits of around 10 to 30 neurons. By the middle of the second week these growing circuits started to connect, allowing the whole network to synchronize.

Analysis of (synchronized) network activity was performed using intra- and inter-burst parameters. On average, network bursts grew in size (mFr) and burst rates increased, thus reducing IBIs. Wagenaar et al. [5] showed similar trends in the majority of cultured networks. In their experiments the median of array-wide spike rate and bursting index increased during the first 3 weeks of culture development. In all models network synchronization mFr tended to rise at later stages (10–13^th^ vDIV) than in cultured networks (usually 7–10^th^ DIV), e.g. [5], [35], [36]. However, Wagenaar et al. showed that smaller cultures usually develop bursting activity at later stages than larger cultures [17]. In this work we did not attempt to analyze spiking activity between the bursts as it largely varies at different levels (e.g. from neuron to neuron, culture to culture) and would require much more experiments.

By increasing the elongation rate in a subset of neurons, thereby generating extensive neuritic structures like in large GABA-ergic cells, young networks also gained shorter graph distances. With such a set of fast growing neurons, network models produced a lower path length *L* and a higher clustering coefficient *C*, resulting in a more effective small-world structure (higher *S*). Effectively, these neurons acted as small hubs as supported by the results of a recent experimental study by Bonifazi et al. [37]. These reduced distances promoted the development of bursts only in field-guided networks. This effect may be explained in the following way. At early developmental stages of simulated networks, neurons have relatively small and sparse neurite arborizations. This reduces the probability of any growing axon to encounter other neurons unless axons are guided in their direction. Therefore, in randomly growing models, the presence of large GABA-ergic neurons has little effect on network connectivity and, thus, bursting started around the same virtual day as in models without large GABA-ergic structures. However, in field-guided models the axons of large GABA-ergic neurons did facilitate connection of local circuits, promoting earlier network burst development. A calcium imaging study by Voigt et al. [10] showed that chemically manipulated cultured networks without large GABA-ergic neurons usually did not develop synchronous activity, which supports the hypothesis that the GABA system promotes the early development of synchronized firing in cultured networks. It is generally assumed that GABA-ergic neurons maintain their excitatory function only during the first two weeks, and then develop into inhibitory neurons. This change of role was not incorporated in our models (large GABA-ergic neurons kept their positive synaptic weights during 3 virtual weeks), which might lead to discrepancies between simulated and experimental data. However, other in vitro studies suggested that the GABA-ergic system may also have an excitatory role even in mature neural networks and may regulate neurite outgrowth (e.g. for review see [], [9], [38,39]). This implies that differences in the development of firing patterns might also be caused by GABA-mediated outgrowth [10]. However, our models did not cover the mechanisms behind neurite outgrowth in chemically manipulated cultured networks. Rather, we used fixed parameters found in studies on normally developing cortical cells [12] and assigned positive weights to their synapses as suggested by Robertson and Menne [38]. Our results showed that even without GABA-mediated outgrowth, large GABA-ergic neurons have a significant effect on the development of firing patterns in field-guided networks. Compared to the other models these networks generated several properties closely resembling experimental data, i.e. earlier start of bursting, moderate increase of mFr and similar development of IBIs.

The essential role of GABA-ergic neurons in synchronization of neuronal activity in cortical cultures was described by Voigt et al. (2001). Our simulation results support their findings and shed light on the excitatory role of large GABA-ergic neurons in the developmental process of cultured cortical networks. Maybe this is as well applicable to in vivo networks. In particular, our results suggest that large GABA-ergic neurons may play a crucial role in triggering early cortical activity (and thus enhance prenatal brain development).

We concluded that simulated networks with large GABA-ergic neurons and axonal guidance showed better agreement to cultured networks than the other models. Therefore we further focused on these models. It is however important to note that the IBI range can also depend on the (mean Poissonian) rate of synaptic noise. In particular, increasing this rate results in reduction of the IBI range (Gritsun et al. 2011). In this study we used constant mean rates of synaptic noise in all the simulations.

Bundles of neurites growing in parallel were formed in the field-guided networks, as seen in experimental networks. The bundles resulted from axons growing along common paths towards spots with higher somatic density. Randomly growing simulation models showed no bundles; neurites grew independently. In contrast, Zubler and Douglas [14] presented a model where neurite bundles were formed due to mechanical processes that modulated neuronal migration. In their study, bundle formation was influenced directly by increasing the attraction strength between growing neurites, whereas our study assumes an alternative (indirect) mechanism based on chemotactic guidance.

For clarity, it is important to note that our approach was based on the assumption of a rather stationary position of grown neurites which, in experimental reality, requires efficiently strong neuronal adhesion to the surface. Insufficient adhesion to the surface usually leads to clustering in experimental preparations. The above described weak bundling should not be confused with the aggregation that occurs when adhesion degrades and the networks subdivides into well defined clusters “islands”, connected by massive thick axonal bundles [40], [41], [42]. Understanding of bundling mechanisms may also be very helpful in the design of central and peripheral highly selective neural prostheses [43], [44].

### Spatio-temporal propagation of network bursts

Even though the majority of the bursts had their own unique distribution of spiking activity, in all network simulations we observed wave-like propagating patterns with several phases. First, a set of easily excitable neurons with strong interconnections in a local area was activated in a chain-like reaction. Their firing increased background activity to connected neurons, thus facilitating activation of those neurons. This prepared the network for the next phase (see Fig. 7, at about 50 ms after the first activity), the fast propagating main-wave of the network burst. This main wave left behind a large pool of depressed neurons which could not be activated shortly after the burst, which usually led to burst cessation, as shown in a previous study [16]. Part of the neurons in that pool, those with relatively fast recovery, occasionally participated in the following “after-wave” spike chains. These spatio-temporal phases reflected on burst shapes as pre-, main- and post phases, as illustrated in Video S1. After-waves usually occurred near the border of the circular network as a result of stronger local connectivity in this area. Since the neurites of frontier neurons could not grow beyond the space of the network, they were “forced” to grow further along the circular boundary. This resulted in a relatively high neurite density and therefore a higher synaptic density in the network border region. This yielded a relatively high synaptic input to the frontier neurons, giving networks a major feedback pathway along their border. When the “after-wave” phase lasted long enough for the other neurons to recover, a subsequent burst could be generated shortly afterwards, with similar phases. This may explain the mechanism behind the generation of superbursts as in experiments, e.g. [5]. This view is supported by the observation that networks without outgrowth restricted boundary did not generate superbursts.

### Boundary conditions and population size

We analyzed the activity of networks with different population sizes. For all network sizes the higher neurite density at the borders provided stronger connectivity and more possible feedback pathways than in the middle of the network. In larger network propagating burst waves had more freedom to travel through the pools of recovered neurons, particularly in these feedback pathways. This may explain the observations that larger network generated highly variable bursting patterns which incorporated both pre- and post-burst phases whereas small networks of 10,000 neurons showed less variable pre-phases only. The development of a prolonged after-wave phase reached its maximum at earlier developmental stages in larger networks. This effect was characterized by earlier occurrence of bursts as well as super bursts in larger network than in small ones, similar to experimental observations by Wagenaar et al. [5].

It is important to note that developing activity patterns can be influenced by several other factors such as long-term synaptic plasticity [45] and apoptosis, incorporation of which may further improve our simulation model. We have shown that up to three weeks LTP does not yet need to be incorporated to explain burt properties in detail. At later times, it will probably have to. The model described here is a good basis for further explorations in those directions.

## Supporting Information

Appendix S1 S1-1. Growth model parameters. S1-2a. Activity model parameters. S1-2b. Synapse model and parameters.
(DOCX)

Figure S1 **Simulation of neurite morphology in a randomly growing network of 10,000 neurons.** The neuronal somas are indicated in green. For 0.5% of these neurons the neurite structures are shown: axons (black) and dendrites (red). The close-up shows only the somas (marked with green and red dots) and axons sprouting from the red marked neurons.
(TIF)

Video S1 **Visualization of spiking activity in a large network of 50,000 neurons with morphological structure at 12 virtual DIV.** Example of a burst series generated by the field-guided model with large GABA-ergic neurons. Top left: Demonstration of the spatio-temporal propagation of spiking waves. The dots show the spatial location of the firing neurons. The circle illustrates the network boundary. Bottom: Shape of the network bursts, as acquired by the virtual MEA (60 electrodes).
(AVI)
