# Supplementary material for: Growth Dynamics Explain the Development of Spatiotemporal Burst Activity of Young Cultured Neuronal Networks in Detail
Source: PLoS One. 2012 Sep 19;7(9):e43352. doi: 10.1371/journal.pone.0043352 (PMC3447003; doi:10.1371/journal.pone.0043352)
Supplement: Appendix S1 — S1-1. Growth model parameters. S1-2a. Activity model parameters. S1-2b. Synapse model and parameters. (DOCX) [file pone.0043352.s001.docx]

**Appendix S1-1. Growth model parameters.**

Besides the parameters given above, in methods (time step 1 day, number of somas 10000-50000, density 2000/mm^2^, culture radius 1.1-2.5 mm, lattice dimensions: 85000-430000 cells/edge 20 μm , soma radius 6.25 μm) , the following parameters were adopted/used from [13] and [20]..

Angular branching bounds [a_min_, a_max_] = -45; +45 degrees.

Ecitatory neurons (see [13], pages 202-204):

- Axonal growth parameters were F= 0.16 and ν_0_ = 45 μm/day. B_∞_=17.38, E=0.39; S=0; τ = 14 days (F is the competition strength parameter, ν_0_ the initial elongation rate, B_∞_ the asymptotic value of the time integral of branching rate function D(t), E the compettion parameter in branching, S the order dependency in branching and τ the time constant in the exponential function D(t) ).
- Dendritic growth parameters: F=0.39 and ν_0_ = 12 μm/day. B_∞_= 4.75, E=0.5; S=0; τ = 3.7 days.

Inhibitory neurons (see [20]):

- Axons: F= 0.16 and ν_0_ = 40 μm/day. B_∞_=17.38, E=0.39; S=0; τ = 14 days.
- Dendrites: F= 0.74 and ν_0_ = 8 μm/day. B_∞_=1.124, E=0.05; S=0; τ = 3.7 days.

**Appendix S1-2a Activity model parameters** *(see also [26])****.***

The neuronal model equation Izhikevich, [31] has the following form:

 (1)

with the auxiliary after-spike resetting

if *v* ≥ +30mV, then $\left\{\begin{matrix} v_{t}=c \\ u_{t}=u_{t-1}+d \end{matrix}\right.$

*in which*

*v is the* membrane potential of the neuron;

*u is the* membrane recovery variable;

*a* describes the time scale of the recovery variable *u*;

*c* and *d* account for action of high-threshold voltage-gated currents activated during the spike.

This spiking model mimics the behavior of several types of cortical neurons. Excitatory neurons exhibited regular spiking (RS), intrinsically bursting(IB) and chattering (CH) behavior; and inhibitory neurons exhibited fast spiking (FS) and low-threshold spiking (LTS) dynamics. These dynamics correspond to the following settings: (*a_i_; b_i_*) = (0.02; 0.2); (*c_i_; d_i_*) = (-65; 8) + (15;-6)*r_i_* ; and (*a_i_; b_i_*) = (0.02; 0.25) +(0.08;-0.05)*r_i_* (*c_i_; d_i_*) = (-65; 2) were assigned to constants of excitatory and inhibitory neurons respectively, where *r_i_* is a random variable normally distributed on the interval [0,1], and *i* is the neuron index. This choice of *a, b, c* and *d* corresponds to a biologically plausible range, see Izhikevich, [31]. We modeled the intrinsic activation feature in ‘pacemaker’ neurons by setting b to values around 0.26.

**Appendix S1-2b Synapse model and parameters.**

In this simulation model we used dynamic (adapting) synapses. Thus, the synaptic weight range was allowed to vary according to the well known STP model of Gupta and Markram [32,33], which mimics short-term facilitation and depression between heterogeneous (excitatory (E) <->inhibitory (I) ) and homogeneous (E-E or I-I) synapses respectively

The dynamics of synapses over time are defined by

$\frac{dI(t)}{dt}=-\frac{I(t)}{\tau_{syn}}+W\delta(t-t_{sp})$ (2)

where *I* is the synaptic current, which decays exponentially with time constant τ*_syn_* , except when a spike occurs in the pre-synaptic neuron at time *t_sp_*. The time constant τ*_syn_* ranged from 3 to 15 ms throughout different synapses.

Synapse adaptation dynamics were modeled via the following set of equations:

$\begin{matrix} w_{k}=A\cdot B_{k}\cdot y_{k} \\ y_{k}=U+y_{k-1}\cdot(1-U)\cdot\exp(-\Delta_{k-1}/F) \\ B_{k}=1+(B_{k-1}-y_{k-1}\cdot B_{k-1}-1)\cdot\exp(-\Delta_{k-1}/F) \\ y_{1}=U \\ B_{1}=1 \end{matrix}$ (3)

where the notation *w_k_* denotes one synaptic weight from W for the *k^-th^* spike, *y* is the running variable for synaptic utilization and B is the running variable for synaptic availability, with *y* and B*=* [0, 1]. The constants *U*, ∆ and F represent the release probability for the first spike, the depression time constant and facilitation time constant, respectively. We used experimental data for U, ∆ and F parameters from [32,33] for all four synapse types, see table 1 below. Authors have shown that these settings lead to biologically plausible synapse models.

*Table 1 E: excitatory, I: inhibitory*

|  | U | ∆ (sec) | F (sec) |
| --- | --- | --- | --- |
| EE | 0.59 | 0.813 | 0 |
| EI | 0.049 | 0.399 | 1.797 |
| IE | 0.16 | 0.045 | 0.376 |
| II | 0.25 | 0.706 | 0.021 |
