# Supplementary figures and images for: Growth Dynamics Explain the Development of Spatiotemporal Burst Activity of Young Cultured Neuronal Networks in Detail
Source: PLoS One. 2012 Sep 19;7(9):e43352. doi: 10.1371/journal.pone.0043352 (PMC3447003; doi:10.1371/journal.pone.0043352)

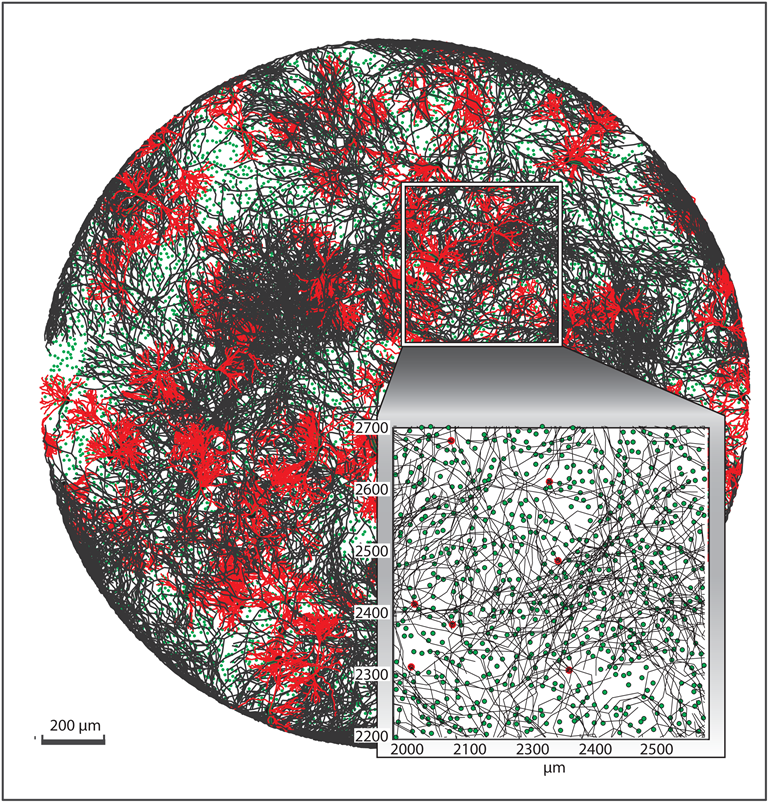

Supplement: Figure S1 — Simulation of neurite morphology in a randomly growing network of 10,000 neurons. The neuronal somas are indicated in green. For 0.5% of these neurons the neurite structures are shown: axons (black) and dendrites (red). The close-up shows only the somas (marked with green and red dots) and axons sprouting from the red marked neurons. (TIF) [file pone.0043352.s002.tif]
